# Interproximal accuracy of CAD/CAM machine cut, machine bent and manually bent retainers: An in vitro comparison

**DOI:** 10.1007/s00056-025-00602-0

**Published:** 2025-07-30

**Authors:** Katharina Klaus, Jan Dirk Pollmeier, Sabine Ruf

**Affiliations:** 1 Department of Orthodontics, Justus Liebig University Giessen, Giessen, Germany Schlangenzahl 14, 35392; 2 Private Orthodontic Practice, Oldenburg, Germany Buergerfelder Str. 2, 26127

**Keywords:** Fixed orthodontic appliances, Lingual fixed retainer, Accuracy, Computer-aided design/computer-aided manufacturing, Digital orthodontics, Festsitzende kieferorthopädische Apparaturen, Festsitzender Lingualretainer, Genauigkeit, Computergestütztes Design/computergestützte Fertigung, Digitale Kieferorthopädie

## Abstract

**Aim:**

The purpose of the present study was to compare the interproximal accuracy (IA) of two different CAD/CAM (computer-aided design/computer-aided manufacturing) manufactured and conventionally bent retainers.

**Materials and methods:**

On 20 digitized debonding casts (10 upper, 10 lower), a canine-to-canine retainer was designed using two different CAD/CAM software: OnyxCeph^®^ (Image Instruments, Chemnitz, Germany) and FixR™ (YOAT Corp., Lynnwood, WA, USA). Each design was bent using the Bender II™ retainer bending machine (YOAT Corp.) and 3‑ or 6‑stranded twistflex wires (0.018-inch Dentaflex^®^, Dentaurum, Ispringen, Germany), respectively. For each cast, one CAD/CAM machine cut retainer (Memotain^®^, SCHEU DENTAL custom-made, Hilden, Germany) was fabricated and one retainer was bent manually by a dental technician using the 6‑stranded wire. All retainers were passively waxed on the dental casts and digitized using a laboratory scanner (Atos, Carl Zeiss IQS Deutschland, Oberkochen, Germany). For each of the five interproximal areas, IA was measured (1) as vertical distance between retainer and the reference retainer plane and (2) as horizontal distance between the interproximal reference point and the retainer in millimeters. For both directions, it was assumed that the smaller the distance, the higher the interproximal accuracy of the retainer. Statistical analysis was performed using Mann–Whitney and Kruskal–Wallis tests.

**Results:**

Considering the vertical deviation, significant differences of interproximal accuracy were seen between the jaws (upper 0.25 mm > lower 0.18 mm, *p* < 0.001), planning software (FixR 0.25 mm > OnyxCeph 0.21 mm, *p* = 0.03) and settings (upper jaw: CAD/CAM bent [FixR] 0.34–0.32 mm > manually bent 0.27 mm > CAD/CAM bent [Onyx] 0.23–0.18 mm > CAD/CAM cut 0.17 mm, *p* = 0.001; lower jaw: CAD/CAM bent [FixR 3‑stranded and Onyx] 0.22–0.21 mm > manually bent 0.18 mm > CAD/CAM bent [FixR 6‑stranded] 0.13 mm > CAD/CAM cut 0.10 mm, *p* = 0.004). For horizontal deviation, significant differences were observed between the jaws (upper 0.97 mm > lower 0.66 mm, *p* < 0.001), planning software (OnyxCeph 0.90 mm > FixR 0.72 mm, *p* < 0.001), settings (upper jaw: CAD/CAM bent [Onyx] = CAD/CAM cut 1.16–1.10 mm > CAD/CAM bent [FixR] 0.96–0.89 mm > manually bent 0.76 mm, *p* < 0.001; lower jaw: CAD/CAM bent [Onyx] 0.84–0.71 mm > CAD/CAM cut 0.66 mm = CAD/CAM bent [FixR] 0.66–0.63 mm > manually bent 0.51 mm, *p* < 0.001) and location of the interdental area in the upper jaw (13–12 = 22–23 > 22–21 > 11–21 > 12–11, *p* < 0.001). The wire quality had no significant impact in either of the dimensions.

**Conclusion:**

Interproximal accuracy in the vertical plane was achieved best by CAD/CAM cut retainers, while horizontally, manually bent retainers were the most accurate. Overall, the accuracy of lower retainers seems clinically acceptable for their use in the mandible irrespective of the manufacturing method, whereas maxillary retainers presented a larger variability especially in the more distal interproximal areas.

**Supplementary Information:**

The online version of this article (https://doi.org/10.1007/s00056-025-00602-0) contains supplementary material, which is available to authorized users.

## Abstract (de)

**Ziel:**

Ziel der vorliegenden Studie war es, die interproximale Genauigkeit (IA) von 2 verschiedenen CAD/CAM-gefertigten und konventionell gebogenen Retainern zu vergleichen.

**Materialien und Methoden:**

Auf 20 digitalisierten Debonding-Modellen (10 Oberkiefer, 10 Unterkiefer) wurde mit 2 verschiedenen CAD/CAM-Softwareprogrammen ein Retainer von Eckzahn zu Eckzahn geplant: OnyxCeph^®^ (Image Instruments, Chemnitz, Deutschland) und FixR™ (YOAT Corp., Lynnwood/WA, USA). Jedes Design wurde mit der Bender II™ Retainer-Biegemaschine (YOAT Corp.) und 3‑ bzw. 6‑strängigen Twistflex-Drähten (0,018-Zoll Dentaflex^®^, Dentaurum, Ispringen, Deutschland) gebogen. Für jedes Modell wurde ein CAD/CAM-gefräster Retainer (Memotain^®^, SCHEU DENTAL custom-made, Hilden, Deutschland) hergestellt und ein Retainer wurde von einem Zahntechniker manuell mit dem 6‑strängigen Draht gebogen. Alle Retainer wurden passiv auf die Zahnmodelle gewachst und mit einem Laborscanner (Atos, Carl Zeiss IQS Deutschland, Oberkochen, Deutschland) digitalisiert. Für jeden der 5 interproximalen Bereiche wurde IA (1) als vertikaler Abstand zwischen Retainer und der Retainerreferenzebene und (2) als horizontaler Abstand zwischen dem interproximalen Referenzpunkt und dem Retainer in Millimetern gemessen. Für beide Richtungen wurde angenommen, dass die interproximale Genauigkeit des Retainers umso höher ist, je kleiner der Abstand ist. Die statistische Analyse wurde mit dem Mann-Whitney- und dem Kruskal-Wallis-Test durchgeführt.

**Ergebnisse:**

Bei der vertikalen Abweichung wurden signifikante Unterschiede in der interproximalen Genauigkeit zwischen den Kiefern (Oberkiefer 0,25 mm > Unterkiefer 0,18 mm, *p* < 0,001), der Planungssoftware (FixR 0,25 mm > OnyxCeph 0,21 mm, *p* = 0,03) und den Konfigurationen festgestellt (Oberkiefer: CAD/CAM gebogen [FixR] 0,34‑0,32 mm > manuell gebogen 0,27 mm > CAD/CAM gebogen [Onyx] 0,23‑0,18 mm > CAD/CAM geschnitten 0,17 mm, *p* = 0,001; Unterkiefer: CAD/CAM gebogen [FixR 3‑fach verseilt und Onyx] 0,22‑0,21 mm > manuell gebogen 0,18 mm > CAD/CAM gebogen [FixR 6‑fach verseilt] 0,13 mm > CAD/CAM geschnitten 0,10 mm, *p* = 0,004). Bei der horizontalen Abweichung wurden signifikante Unterschiede zwischen den Kiefern (Oberkiefer 0,97 mm > Unterkiefer 0,66 mm, *p* < 0,001), der Planungssoftware (OnyxCeph 0,90 mm > FixR 0,72 mm, *p* < 0,001) und den Einstellungen (Oberkiefer: CAD/CAM gebogen [Onyx] = CAD/CAM geschnitten 1,16–1,10 mm > CAD/CAM gebogen [FixR] 0,96–0,89 mm > manuell gebogen 0,76 mm, *p* < 0,001; Unterkiefer: CAD/CAM gebogen [Onyx] 0,84–0,71 mm > CAD/CAM geschnitten 0,66 mm = CAD/CAM gebogen [FixR] 0,66‑0,63 mm > manuell gebogen 0,51 mm, *p* < 0,001) und Lokalisation des Interdentalbereichs im Oberkiefer beobachtet (13–12 = 22–23 > 22–21 > 11–21 > 12–11, *p* <0,001). Die Drahtqualität hatte keinen signifikanten Einfluss auf eine der beiden Dimensionen.

**Schlussfolgerung:**

Die interproximale Genauigkeit in der vertikalen Ebene wurde am besten von CAD/CAM-geschnittenen Retainern erreicht, während in der horizontalen Ebene manuell gebogene Retainer die höchste Genauigkeit aufwiesen. Insgesamt scheint die Genauigkeit von Retainern für den Unterkiefer unabhängig von der Herstellungsmethode klinisch akzeptabel zu sein, während Retainer für den Oberkiefer eine größere Variabilität aufweisen, insbesondere in den distalen interproximalen Bereichen.

## Introduction

Long-term retention of orthodontic treatment results is necessary to counteract alignment relapse tendencies, but also to avoid occlusal changes due to physiological aging and late facial growth [12]. Orthodontic retention can be achieved either using removable appliances or by the placement of bonded lingual retainers. The latest update of the Cochrane review found only marginally better stability with fixed retainers compared to full- or part-time worn removable retainers, a finding that is constrained by a low level of evidence and a short-term follow-up of only 12 months [13]. While patients prefer fixed retainers more than removable retainers in terms of acceptance, compliance, comfort and speech [13], preferences of orthodontists vary substantially, as suggested by questionnaire-based studies of different countries around the world [12]. Practitioners in the United States, Canada, Australia, and New Zealand favor removable appliances in the maxilla and fixed retainers in the mandible, while orthodontists in Europe prefer fixed retainers for both jaws [3, 11, 14, 16, 17, 24, 25, 27]. Whereas fixed retainers in the mandible are mostly bonded to all six anterior teeth from canine to canine (33–43), varying extensions are reported for the maxillary teeth, ranging from six teeth (13–23) over four teeth (12–22) to short retainers only bonded to the central incisors (11–21) [3, 11, 14, 16, 27]. Present knowledge regarding fixed retainers was largely influenced by the work of Björn U. Zachrisson, who started reporting his experience with bonded retainers in 1977 [28] and in 2015 finally recommended the use of 12–22 retainers in the maxilla and 33–43 retainers in the mandible, both bent of 5‑stranded coaxial wire (five strands wrapped around one central strand) with 0.0215-inch diameter [30]. Nearly around the same time, lingual retainers created by computer-aided design/computer-aided manufacturing (CAD/CAM) were introduced [10, 26], which are planned either on digitized plaster casts or intraoral scans and subsequently laser-cut or milled out of a material blank. Alternatively, CAD/CAM retainers can be fabricated by laser-melting or 3D-printing, but to date, there is no sufficient evidence supporting the clinical use of the latter [19, 23]. However, CAD/CAM cut or milled retainers have been intensively investigated during the last decade and show highly precise positioning accuracies [9, 26] as well as comparable stability values to conventional multistranded fixed retainers [13, 18]. Nevertheless, the additional requirement for working plaster casts or scans, the manufacturing and shipping time associated with these processes and their related costs can be seen as common disadvantages of both conventional and CAD/CAM cut or milled retainers. Recently, a retainer bending machine (Bender II, YOAT Corporation, Lynnwood, WA, USA) offers a combination of the two methods with computer-aided design (CAD) of the retainer based on an intraoral scan and subsequently mechanical bending of the retainer (CAM) from round multistranded or straight rectangular wire within 5 min. However, to date, evidence regarding the performance of this retainer manufacturing method is scarce. One research group conducted a randomized, controlled trail comparing CAD/CAM bent, manually bent and chairside adapted retainers and found significantly less relapse in the CAD/CAM bent group but no significant difference in failure rates [22]. Two in vitro studies aimed to investigate the positioning accuracy of CAD/CAM bent retainers: the first compared machine bent retainers of round 3‑stranded twistflex and straight rectangular wire and found a slightly larger vertical distance between the retainer and interproximal contact points for rectangular retainers [5]. The second compared CAD/CAM cut with CAD/CAM and manually bent retainers of 3‑stranded twistflex wire and revealed a significantly lower interproximal accuracy measured in the horizontal plane for CAD/CAM bent retainers in the upper jaw, while in the lower jaw, the same tendency was seen but did not reach statistical significance [8]. The latter also investigated the vertical deviation of the three retainer types for the upper and lower jaw, but these measurements were not performed in the interproximal area [8]. Precise interproximal accuracy in the horizontal and vertical dimension is mandatory for fixed retainers, as horizontally poor fitting retainers lead to patient discomfort and increase the risk of wire distortion by hard food components, possibly leading to activation or even breakage of the wire. Vertical deviations especially in the maxillary arch increase the challenge to insert the retainer outside the occlusal range of the lower incisors [10].

However, to date research on CAD/CAM bent retainers focused solely on 3‑stranded twistflex wire of 0.5 mm diameter and is limited to one CAD planning software [5, 8]. Therefore, the aim of the present study was to analyze the interproximal accuracy of CAD/CAM machine cut, manually bent and CAD/CAM machine bent retainers in the vertical and horizontal plane. The primary research question was whether the interproximal accuracy differs among retainers manufactured using different methods. Additional questions addressed whether there are differences in accuracy between the maxilla and mandible, among different interproximal areas along the retainer, and among CAD/CAM machine bent retainers with different wire qualities and various planning software.

## Materials and methods

**Fig. 1:**
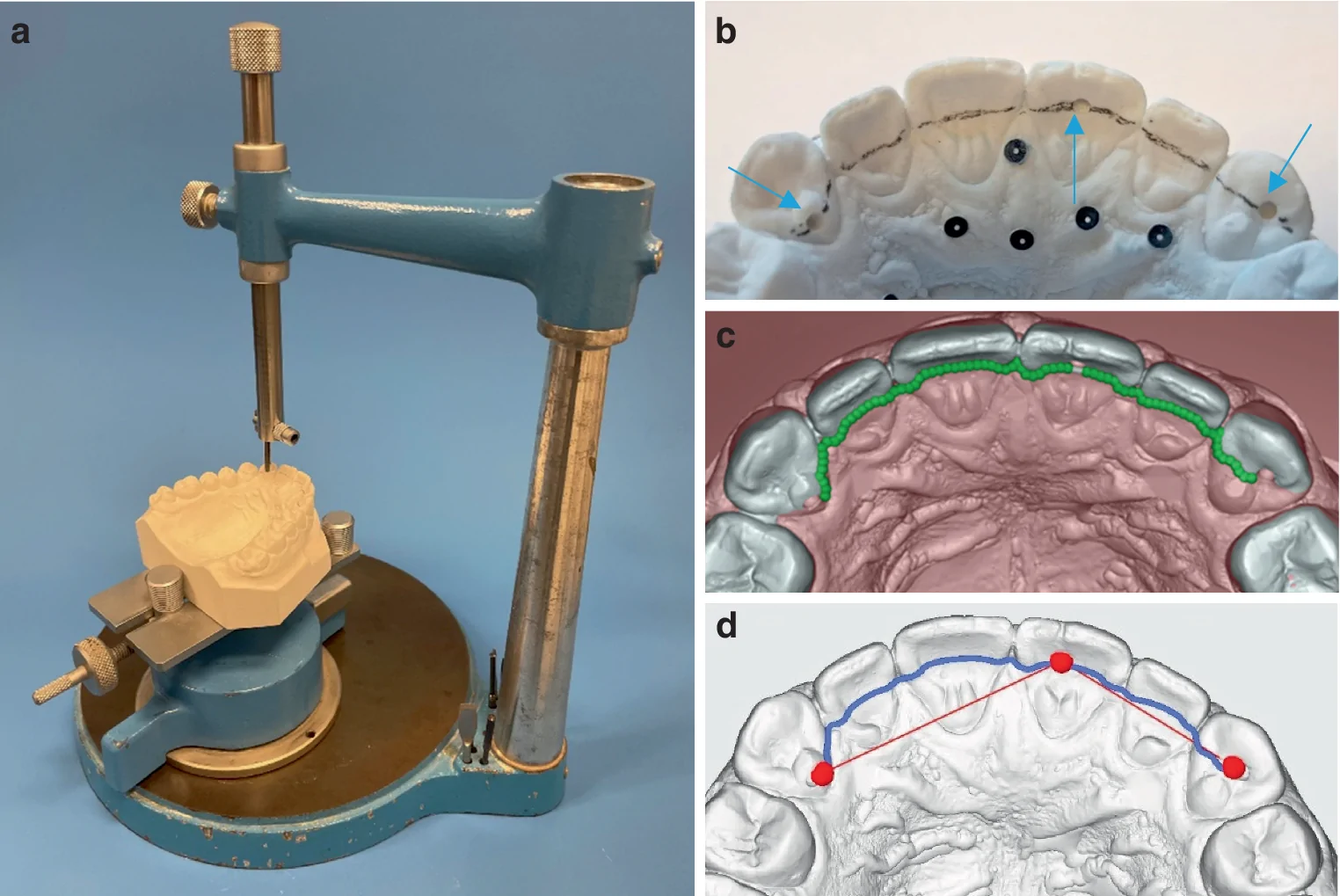
Workflow of retainer design. **a** Determination of the retainer plane on the physical model. **b** Marking the retainer plane with small dimples on teeth 13, 21, and 23 (blue arrows). **c** Retainer design using the FixR™ software. **d** Retainer design using the OnxyCeph^®^ software Ablauf des Retainerdesigns. **a** Festlegung der Retainerebene am physischen Modell. **b** Markierung der Retainerebene mittels kleiner Vertiefungen an den Zähnen 13, 21 und 23 (blaue Pfeile). **c** Retainerdesign mittels der Software FixR™. **d** Retainerdesign mittels der Software OnyxCeph^®^

Maxillary and mandibular plaster casts (*n* = 20) from 10 patients who completed their multibracket appliance treatment in 2022 at the Department of Orthodontics, Justus Liebig University Giessen, Germany, were chosen for this study. The selection criteria included the following: naturally healthy canines, lateral and central incisors free from adhesive or prosthetic restorations, no crowding or spacing issues, and a diverse range of individual tooth crown anatomies, particularly in the maxilla. The debonding plaster casts were duplicated, and the retainer plane was determined using a surveyor (VG‑1, Degussa, formerly Hanau, Germany), which was then marked on the lingual surfaces of the canines, as well as the lateral and central incisors in both jaws (Fig. 1a). Subsequently, small dimples were drilled into the distal lingual surface of the maxillary and mandibular canines and into the middle lingual surface of tooth 21 (maxilla) and tooth 31 (mandible) along the retainer planes, utilizing a small round diamond bur (Fig. 1b). All 20 casts were digitized with a laboratory scanner (Atos, GOM Zeiss Metrology, Oberkochen, Germany) and saved as standard tessellation language (STL) files.

### Manufacturing of CAD/CAM machine bent retainers

The generated STL files were then imported into the FixR™ software (YOAT Corp., Lynnwood, WA, USA) and to the OnyxCeph^®^ software (Image Instruments, Chemnitz, Germany), where the retainers were designed along the plane defined by the three dimples while avoiding the inner of the dimples (Fig. 1c and d).

The Bender II™ retainer bending machine (YOAT Corp., Lynnwood, WA, USA) was calibrated for a 3-stranded twistflex wire of 0.018-inch diameter (Dentaflex®, Dentaurum, Ispringen, Germany) according to the manufacturer’s instructions. Subsequently, all retainer designs were bent using the 3‑stranded twistflex wire. Afterwards, the Bender II™ was calibrated for a 6-stranded coaxial twistflex wire of the same diameter (Dentaflex^®^, Dentaurum, Ispringen, Germany) and all retainer designs were bent using the 6‑stranded coaxial wire.

### Manufacturing of CAD/CAM machine cut retainers

Memotain^®^ retainers (SCHEU DENTAL custom-made, Hilden, Germany) were ordered based on the STL files of the 20 models. Instructions regarding the course of the retainer along the plane marked by the dimples, bypassing the inner area of them, were communicated to the company both verbally and in written form.

### Manufacturing of manually bent retainers

Based on the duplicated plaster cast models, 20 retainers were manually bent using the 0.018 inch 6‑stranded coaxial twistflex wire (Dentaflex®, Dentaurum, Ispringen, Germany), as this type of retainer represents the current clinical standard at the Department of Orthodontics. The same instructions regarding the course of the retainer as mentioned above were communicated both verbally and in writing. All retainers were bent by the same dental technician who has 17 years of experience in manufacturing orthodontic appliances.

### Measurement of interproximal accuracy

**Fig. 2:**
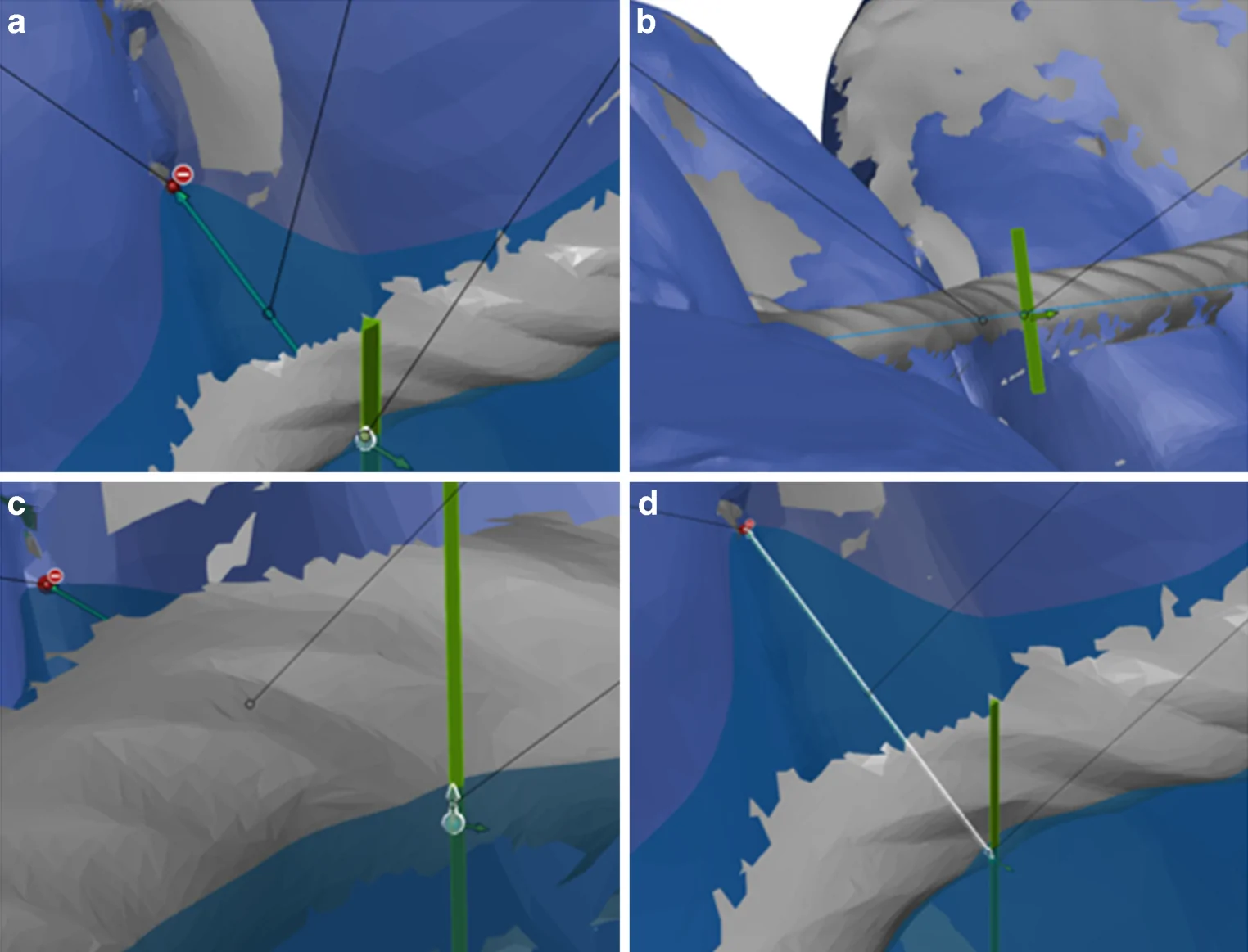
Visualization of measurements. **a** Determination of the interproximal measurement point (red) as intersection of the retainer reference plane (light blue) with the interproximal mesh of the reference standard tesselation language (STL) file. **b** Probing the retainer (green bar) from palatal parallel to the reference plane (light blue). **c** Vertical measurement: distance from the probing point (white) to the retainer reference plane (blue). **d** Horizontal measurement: distance from the probing point to the interproximal reference point. Please note that the thickness of the retainer was subtracted from the measured value Visualisierung der Messungen. **a** Festlegung des interdentalen Messpunktes (rot) als Schnittpunkt zwischen der Retainer-Referenzebene (hellblau) und des interdentalen Mesh der Referenz-STL(„standard tesselation language“)-Datei. **b** Antasten des Retainers (grüner Balken) von palatinal parallel zur Referenzebene (hellblau). **c** Vertikale Messung: Lotrechte Distanz zwischen Tastpunkt (weiß) und Referenzebene (blau). **d** Horizontale Messung: Distanz zwischen Tastpunkt und interdentalem Messpunkt. Die Dicke des Retainers wurde im Anschluss an die Messung vom jeweiligen Messwert abgezogen

**Fig. 3:**
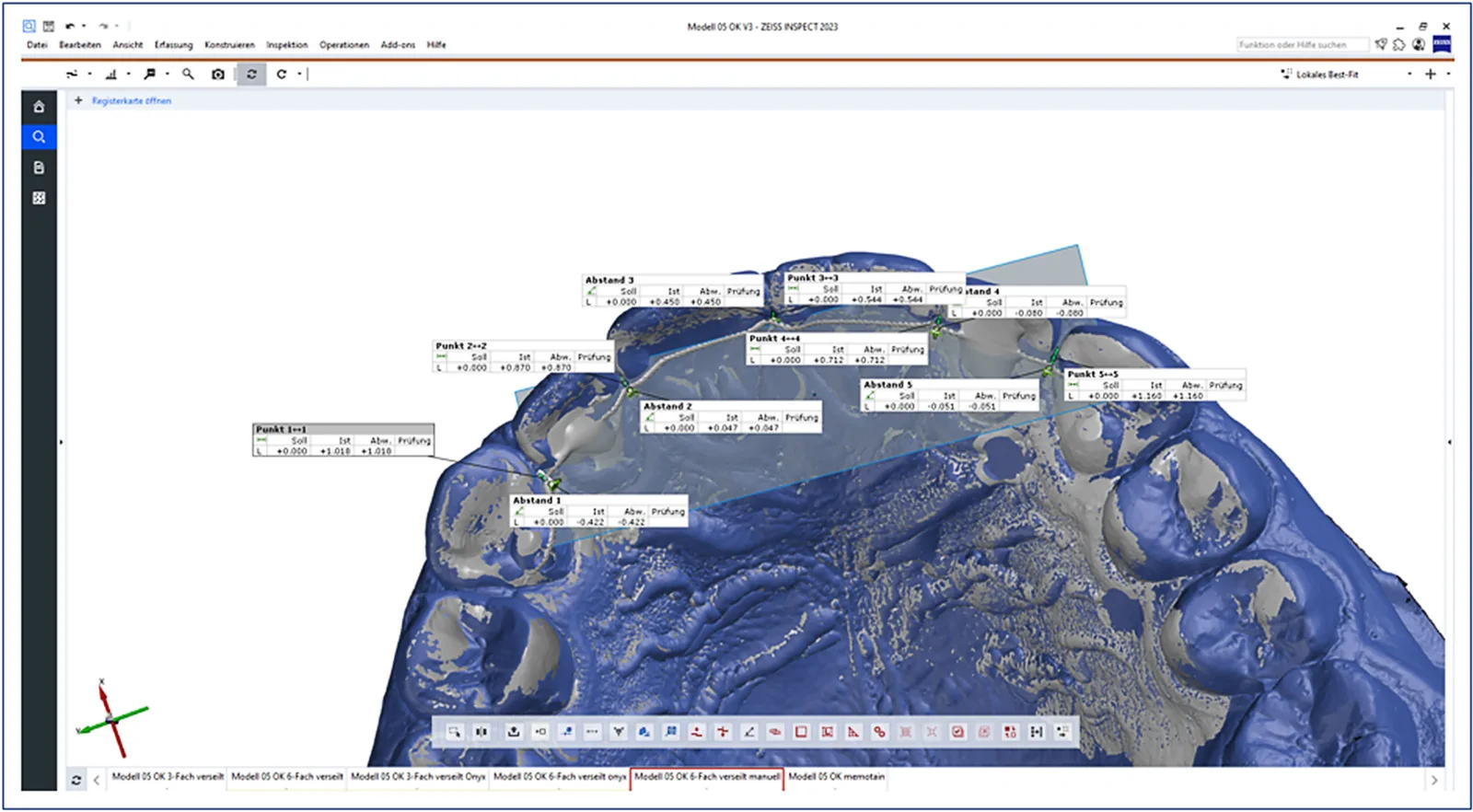
Screenshot of the computerized measurements in Zeiss Optical 3D pro Screenshot der computergestützten Messungen in Zeiss Optical 3D pro

All fabricated retainers were sequentially fixed passively at two points (typically at the lingual surfaces of the lateral incisors) on their respective model using wax. For digitization, the wire surfaces were matted with scanning spray (Cerec Optispray, Sirona Dental Systems, Bensheim, Germany), and each model along with each retainer (*n* = 120) was digitized with a laboratory scanner (Atos, Carl Zeiss IQS Deutschland, Oberkochen, Germany). Using the software Zeiss Inspect Optical 3D Pro (Carl Zeiss IQS Deutschland, Braunschweig, Germany), the model without a retainer was imported as reference model and the respective retainer reference plane was created based on the three dimples. For each interproximal space along the course of the retainer (*n* = 5), the interproximal measurement point was defined as the deepest point where the reference plane intersects with the interproximal mesh of the standard tesselation language (STL) file (Fig. 2a). Five interproximal planes perpendicular to the reference plane were created. Afterwards, the files of the digitized retainers were imported and a batchwise best-fit superimposition was undertaken using the occlusal surfaces of premolars and molars, anterior palate (maxilla) and tongue base (mandible) as unchanged reference structures. The interproximal accuracy was measured in vertical and horizontal directions by determination of measurement principles, which were automatically adapted to each retainer model due to the batchwise superimposition. Using the “probing” function, the retainers were probed from palatal/lingual along each of the five interproximal planes and parallel to the retainer plane (Fig. 2b). For vertical accuracy, the distance of the probing point on the retainer and the retainer plane was recorded (Fig. 2c). For horizontal accuracy, the value was obtained by subtracting the average thickness of the retainer (0.45 mm for the twistflex wires, 0.4 mm for Memotain^®^) from the distance between the probing point and the interproximal point (Fig. 2d). For both directions, it was assumed that the smaller the distance, the higher the interproximal accuracy of the retainer. Figure 3 gives an overview of the computerized measurements.

### Statistical analysis

Besides descriptive data analysis using minimum, quartiles and maximum, comparisons of the factors jaw (2 factor levels), settings (6), wire quality (2), planning software (2), and location (5) in relation to the interproximal accuracy in vertical and horizontal direction were conducted as nonparametric tests (Mann–Whitney and Kruskal–Wallis), determining that a *p*-value of 0.05 or less indicates a statistically significant difference. All tests were performed exploratively: (1) the test for settings and location were conducted separately for maxilla and mandible, (2) for all other comparisons, the factor levels not subject to testing were aggregated. Because of the exploratory character of the analyses, it was decided not to lower the nominal alpha (α) level for undertaking multiple testing. In addition to the statistical tests, standardized effect size measures were calculated: r for the Mann–Whitney test [7], which is interpreted as follows [4]: r ≥ 0.1: weak effect, r ≥ 0.3: medium effect, r ≥ 0.5: strong effect and for the Kruskal–Wallis test; η^2^ (eta^2^) was calculated [21] and interpreted as follows [4]: η^2^ ≥ 0.01: weak effect, η^2^ ≥ 0.06: medium effect, η^2^ ≥ 0.14: strong effect. For the different settings, pairwise comparisons were undertaken using the Dunn test adjusted to Hochberg. All statistical tests were conducted with Stata software version 16.1 (StataCorp LLC, TX, USA). A post hoc sensitivity analysis was carried out using GPower version 3.1.9.7. (HHU Düsseldorf, Düsseldorf, Germany).

## Results

### Vertical interproximal accuracy

The median vertical distance between the retainers and the retainer reference plane was 0.25 mm in the maxilla and 0.18 mm in the mandible. The Mann–Whitney test indicated a significant difference between the jaws (*p* = 0.0001, r = 0.156), but with a weak effect (Table 1).

**Table 1:** Descriptive measures of vertical interproximal distances for the factors jaw, wire qualities, and planning software Deskriptive Kennzahlen der vertikalen interdentalen Distanzen für die Faktoren Kiefer, Drahtqualität und Planungssoftware

	Minimum	1st quartile	Median	3rd quartile	Maximum	*p*-value	r
Maxilla	0.00	0.10	0.25	0.42	1.81	0.0001	0.16
Mandible	0.00	0.07	0.18	0.30	0.99		
3‑stranded	0.00	0.14	0.23	0.39	1.81	0.0845	0.09
6‑stranded	0.00	0.08	0.22	0.36	0.96		
FixR	0.00	0.12	0.25	0.40	1.39	0.0287	0.11
Onyx	0.00	0.10	0.21	0.39	1.81		

**Fig. 4:**
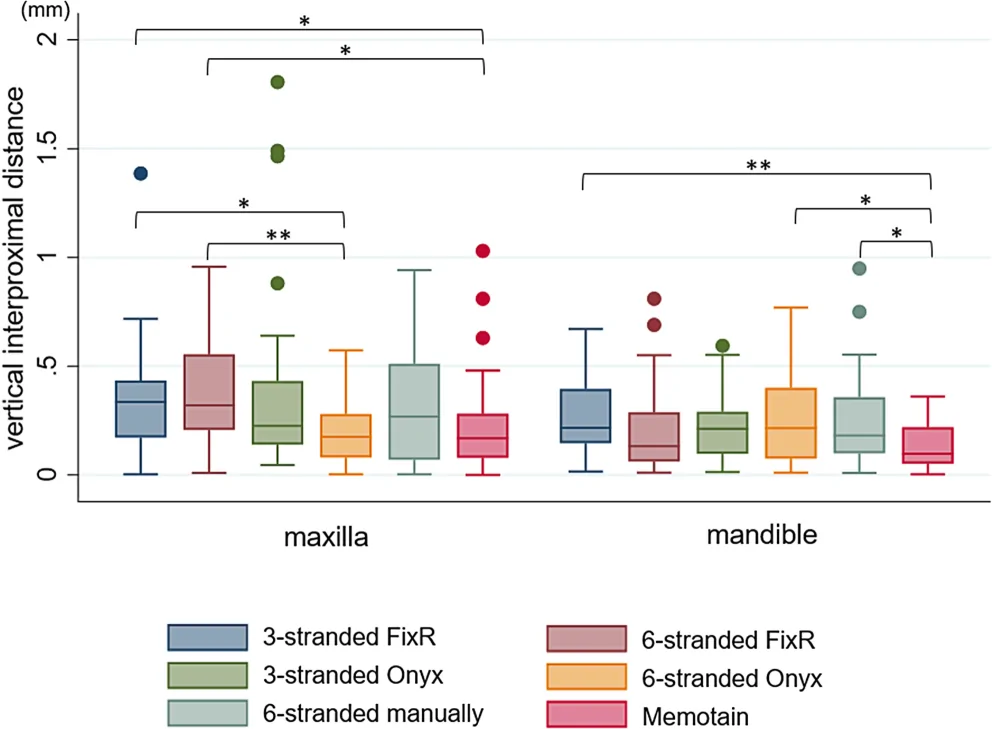
Vertical interproximal distances for the different retainer settings. Significant pairwise comparisons are denoted by asterisks. * *p* < 0.05, ** *p* < 0.01. The corresponding *p*-values and effect size estimates of all pairwise comparisons are given in the supplementary table Vertikale interdentale Distanzen für die verschiedenen Retainersettings. Signifikante paarweise Vergleiche sind mit Sternchen markiert. * *p* < 0,05, ** *p* < 0,01. Die dazugehörigen *p*-Werte und Effektgrößen aller paarweisen Vergleiche stehen als Zusatzmaterial zur Verfügung

Between the different retainer settings, significant differences with weak effect sizes were obtained in the maxilla (*p* = 0.0011, η^2^ = 0.05) and the mandible (*p* = 0.004, η^2^ = 0.04). Regarding the median values, in both jaws, the Memotain^®^ retainer had the highest vertical accuracy, while the lowest accuracy was seen for CAD/CAM machine bent retainers from 3‑stranded wire designed by the FixR software (Table 2; Fig. 4).

**Table 2:** Descriptive measures of vertical interproximal distances for the different retainer settings in the maxilla and mandible Deskriptive Kennzahlen der vertikalen interdentalen Distanzen für die unterschiedlichen Retainersettings im Ober- und Unterkiefer

*Maxilla*	*Minimum*	*1st quartile*	*Median*	*3rd quartile*	*Maximum*	*p‑value*	*η*^*2*^
FixR 3‑stranded	0.00	0.17	0.34	0.43	1.39	0.0011	0.05
FixR 6‑stranded	0.01	0.21	0.32	0.55	0.96		
Onyx 3‑stranded	0.05	0.14	0.23	0.43	1.81		
Onyx 6‑stranded	0.00	0.08	0.18	0.28	0.57		
Manually 6‑stranded	0.00	0.07	0.27	0.51	0.94		
Memotain	0.00	0.08	0.17	0.28	1.03		
*Mandible*	*Minimum*	*1st quartile*	*Median*	*3rd quartile*	*Maximum*	*p‑value*	*η*^*2*^
FixR 3‑stranded	0.02	0.15	0.22	0.40	0.67	0.0040	0.04
FixR 6‑stranded	0.01	0.06	0.13	0.29	0.81		
Onyx 3‑stranded	0.01	0.10	0.21	0.29	0.59		
Onyx 6‑stranded	0.01	0.08	0.22	0.40	0.77		
Manually 6‑stranded	0.01	0.10	0.18	0.36	0.95		
Memotain	0.00	0.05	0.10	0.22	0.36

**Table 3:** Descriptive measures of horizontal interproximal distances for the factors jaw, wire qualities and planning software Deskriptive Kennzahlen der horizontalen interdentalen Distanzen für die Faktoren Kiefer, Drahtqualität und Planungssoftware

	Minimum	1st quartile	Median	3rd quartile	Maximum	*p*-value	r
Maxilla	0.23	0.72	0.97	1.26	3.08	< 0.0001	0.41
Mandible	0.00	0.53	0.66	0.85	2.43		
3‑stranded	0.15	0.62	0.77	1.07	3.08	0.4051	0.04
6‑stranded	0.31	0.63	0.83	1.13	2.63		
FixR	0.25	0.58	0.72	1.00	2.24	< 0.0001	0.25
Onyx	0.15	0.68	0.90	1.29	3.08

**Fig. 5:**
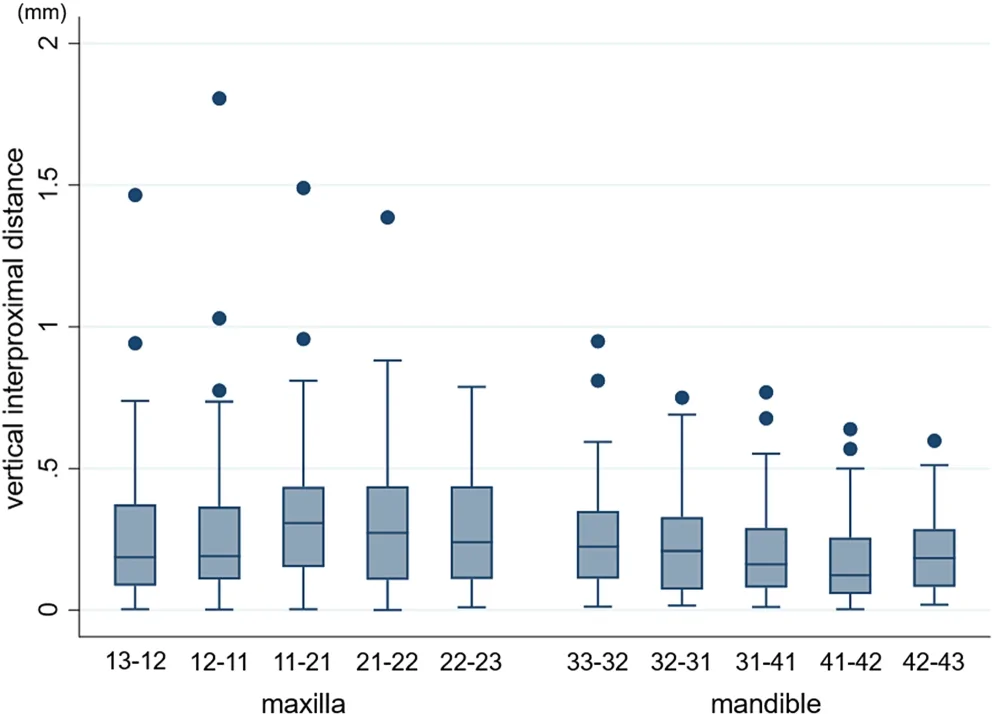
Vertical interproximal distances of aggregated retainer settings measured for the different interproximal areas Vertikale interdentale Distanzen der aggregierten Retainersettings im Bereich der verschiedenen Interdentalräume

Taking the location of the interproximal areas into account, no statistically significant vertical deviations were observed between the different interproximal areas for neither the maxilla nor for the mandible (maxilla: median 0.19–0.31 mm, *p* = 0.2764, r = 0.01, mandible: median 0.12–0.22 mm, *p* = 0.1433, r = 0.01, Fig. 5).

Considering only the CAD/CAM machine bent retainers, there was no significant difference between the 3‑ and 6‑stranded twistflex wires (median 3‑stranded: 0.23 mm, 6‑stranded: 0.22 mm, *p* = 0.0845, r = 0.09). Comparing the two CAD software, retainers planned by OnyxCeph^®^ had a slightly lower median vertical distance than those planned by FixR™ (median Onyx: 0.21 mm, FixR 0.25 mm, *p* = 0.0287, r = 0.11) which reached statistically significance, but with a weak effect size (Table 1).

### Horizontal interproximal accuracy

The median horizontal distance between the retainers and the interproximal reference points was 0.97 mm in the maxilla and 0.66 mm in the mandible, indicating a statistically significant difference with a medium effect size (*p* < 0.0001, r = 0.41; Table 3).

**Fig. 6:**
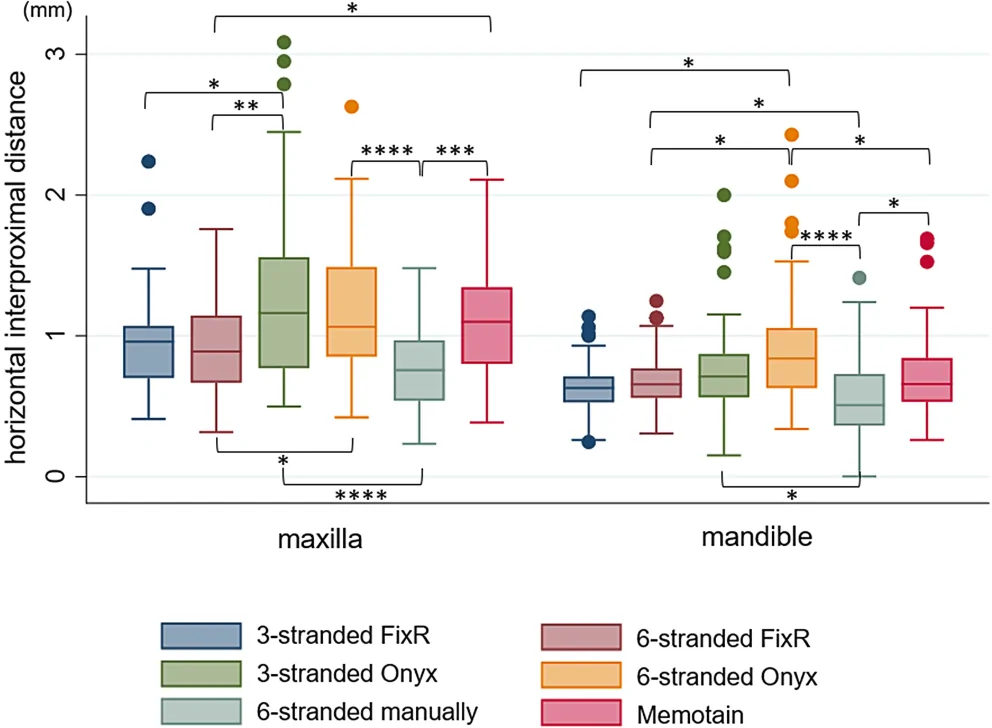
Horizontal interproximal distances for the different retainer settings. Significant pairwise comparisons are denoted by asterisks. * *p* < 0.05, ** *p* < 0.01, *** *p* < 0.001, **** *p* < 0.0001. The corresponding *p*-values and effect size estimates of all pairwise comparisons are given in the supplementary table Horizontale interdentale Distanzen für die verschiedenen Retainersettings. Signifikante paarweise Vergleiche sind mit Sternchen markiert. * *p* < 0,05, ** *p* < 0,01, *** *p* < 0,001, **** *p* < 0,0001. Die dazugehörigen *p*-Werte und Effektgrößen aller paarweisen Vergleiche stehen als Zusatzmaterial zur Verfügung

Significant differences with medium effect sizes between the retainer settings were seen in the maxilla (*p* = 0.0001, η^2^ = 0.11) and the mandible (*p* = 0.0001, η^2^ = 0.10). Regarding the median values, the manually bent retainers in both jaws had the highest horizontal accuracy, while both the CAD/CAM machine cut and bent retainers reached comparable values (Table 4; Fig. 6). Noticeably high outliers were seen in the maxilla (up to 3.08 mm) and also the mandible (up to 2.43 mm) for the CAD/CAM machine bent retainers which had been planned with the OnyxCeph^®^ software.

**Table 4:** Descriptive measures of horizontal interproximal distances for the different retainer settings in the maxilla and mandible Deskriptive Kennzahlen der horizontalen interdentalen Distanzen für die unterschiedlichen Retainersettings im Ober- und Unterkiefer

*Maxilla*	*Minimum*	*1st quartile*	*Median*	*3rd quartile*	*Maximum*	*p‑value*	*η*^*2*^
FixR 3‑stranded	0.41	0.70	0.96	1.07	2.24	0.0001	0.11
FixR 6‑stranded	0.32	0.67	0.89	1.14	1.76		
Onyx 3‑stranded	0.50	0.77	1.16	1.56	3.08		
Onyx 6‑stranded	0.42	0.85	1.06	1.49	2.63		
Manually 6‑stranded	0.23	0.54	0.76	0.97	1.48		
Memotain	0.38	0.80	1.10	1.35	2.11		
*Mandible*	*Minimum*	*1st quartile*	*Median*	*3rd quartile*	*Maximum*	*p‑value*	*η*^*2*^
FixR 3‑stranded	0.25	0.53	0.63	0.71	1.14	0.0001	0.10
FixR 6‑stranded	0.31	0.56	0.66	0.77	1.25		
Onyx 3‑stranded	0.15	0.56	0.71	0.87	2.00		
Onyx 6‑stranded	0.34	0.63	0.84	1.06	2.43		
Manually 6‑stranded	0.00	0.36	0.51	0.73	1.41		
Memotain	0.26	0.53	0.66	0.84	1.69

**Fig. 7:**
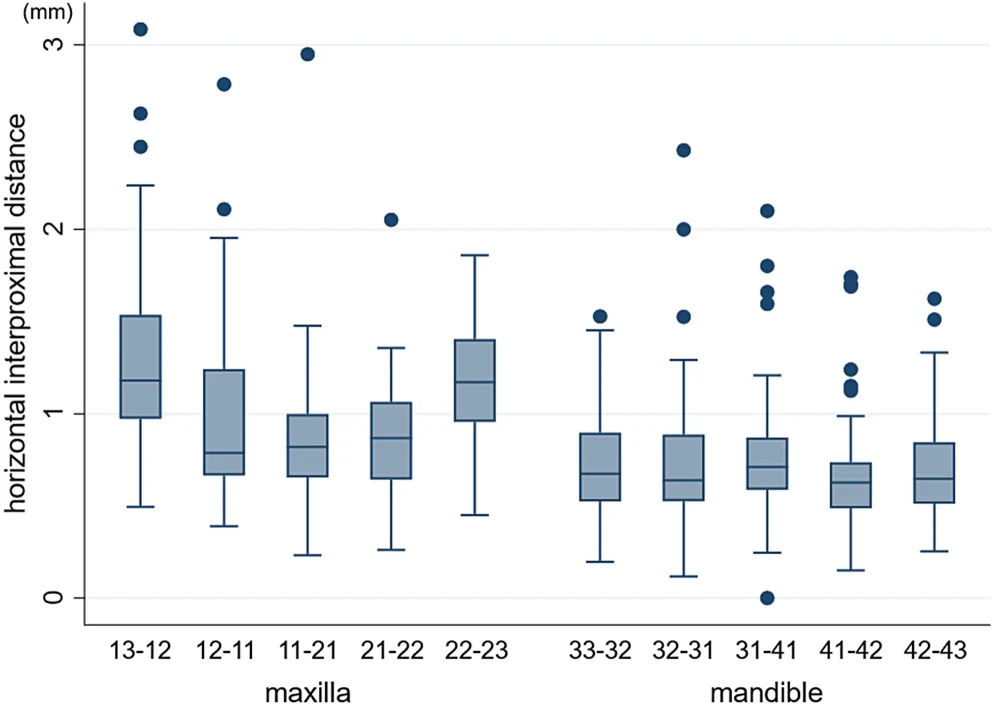
Horizontal interproximal distances of aggregated retainer settings measured for the different interproximal areas Horizontal interdentale Distanzen der aggregierten Retainersettings im Bereich der verschiedenen Interdentalräume

The location of the different interproximal areas in the maxilla had a significant impact with a strong effect size on the measured horizontal interproximal distance (*p* = 0.0001, η^2^ = 0.18), with the largest values obtained between the canines and the lateral incisors (median: 1.18/1.17 mm). The interproximal areas located between the incisors showed comparable values (median 0.79–0.87 mm). For the mandible, no significant differences between the different interproximal areas were found (*p* = 0.1738, η^2^ = 0.01; Fig. 7).

Focusing on the CAD/CAM machine bent retainers, there was no significant difference between the 3‑ and 6‑stranded twistflex wires (median 3‑stranded: 0.77 mm, 6‑stranded: 0.83 mm, *p* = 0.4051, r = 0.04). Examining the two CAD software, retainers planned by OnyxCeph^®^ had a higher median horizontal distance than those planned by FixR™ (median Onyx: 0.90 mm, FixR 0.72 mm, *p* < 0.0001, r = 0.25), resulting in a highly significant difference, but with a weak effect size (Table 1).

## Discussion

The ongoing digitization of orthodontics during the last few decades has led to a substantial shift in clinical routines, but also increased the need for scientific evaluation of newly developed technologies. While the first CAD/CAM milled fabricated lingual retainer was introduced in 2015 [26], approximately 10 years later, their performance was analyzed by systematic reviews and meta-analyses [1, 13]. Given the fact that the Bender was introduced in 2019, scientific evidence is reasonably scarce.

### Measurement methods

The methodology of measuring the horizontal interproximal accuracy applied in the present study has previously been used by Kang et al. [8] and was only slightly adapted to the different measurement software. Despite the fact that Kang et al. [8] showed acceptable reliability values for the methodology, we decided to automatize the measurements by batchwise superimpositions to further reduce the possibility for systematic measurement errors. Because the measuring principles were predefined, all measurements were automated, removing the need for manual reference point identification and, thus, eliminating the necessity for repeated measurements to evaluate potential intraindividual measurement error. Similar to Kang et al. [8], we probed the retainers from the palatal/lingual direction and subtracted the thickness of the retainer (0.45 mm for Dentaflex^®^, 0.4 mm for Memotain^®^) as reported by the manufacturers. This could have led to some marginal inaccuracies, as the real thickness may vary due to manufacturing tolerances and surface changes during bending. Of course, it would be preferable to probe the retainers from the opposite site for direct measurement of the interproximal distance, but this area cannot be satisfactory scanned even by laboratory scanners due to undercuts between the interproximal area and the retainer. Another limitation of the probing direction could be seen in the comparison of round and rectangular retainers: probing retainers from the palatal/lingual direction captures the outmost boundary of round wires, while for the rectangular Memotain^®^, the corresponding midpoint of the 0.4 mm high oral surface was chosen. By subtracting the thickness of the retainers, the interproximal distance to the outmost boundary in tooth direction was recorded, which corresponds to the point of a round wire touching the curved lingual surface. However, a rectangular retainer touches the lingual tooth surface with its lower vestibular edge, therefore, up to 0.2 mm discrimination of the rectangular Memotain^®^ retainer must theoretically be considered. Finally, one could argue that the measured horizontal interproximal distance does not reflect the true horizontal direction, because in retainers with increasing vertical deviation, the distance to the fixed interproximal reference point located on the retainer reference plane increases, too. Nevertheless, a reproducible reference point is mandatory for any measurement and was therefore chosen according to Kang et al. [8]. Especially considering the results of the vertical interproximal measurements, this limitation would not impede the clinical relevance of the obtained values.

### Statistical methods

Due to the nonnormative distribution of the data including considerable outliers, common data transformation strategies failed to achieve a sufficient approximation to normal distribution. Therefore, nonparametric tests were used, which were performed exploratively: the test for settings and location were conducted separately for maxilla and mandible; for all other comparisons, the factor levels not subject to testing were aggregated. One could argue that this potentially violates the assumption of independence for testing; therefore, the tests should be interpreted exploratively. This approach seemed justified given the multitude of factors (and factor levels) to be tested and the explorative nature of the question. To ensure adequate statistical power with the given sample size, a post hoc sensitivity analysis was conducted based on the horizontal interproximal accuracy between the maxilla and mandible respectively between the retainer settings. This analysis showed that with the current group size, an α error of 5% and a power of 80%, medium effect sizes in the population below r = 0.3 can be statistically reliably detected.

### Results

In the vertical dimension, CAD/CAM cut retainers showed the highest interproximal accuracy in both jaws. This could have been expected due to their manufacturing process, as they are cut or milled out of a flat material blank. The present results are in line with previous studies investigating the intraoral positioning accuracy of CAD/CAM cut retainers compared to their planned position [9, 26]. Looking at the retainers bent from a multistranded wire, in the maxilla the CAD/CAM bent retainers designed by OnyxCeph^®^ were the most accurate with median deviations ranging from 0.18 to 0.23 mm, followed by the manually bent (median: 0.27 mm) and the CAD/CAM bent designed by FixR™ retainers (median: 0.32–0.34 mm). The Kruskal–Wallis test revealed these differences to be statistically significant, but with a weak effect size, a finding that was also confirmed in the mandible with even smaller differences between the bent retainers, suggesting that the differences in vertical interproximal accuracy might be statistically significant, but clinically irrelevant. Furthermore, the median vertical deviations of all retainers were smaller than 0.5 mm, a threshold which is commonly mentioned to express clinical relevance [9, 26]. Nevertheless, it should be noted that especially the maxillary CAD/CAM bent retainers made from 3‑stranded wire had the largest maximum vertical deviations of 1.39–1.81 mm, which can certainly be of clinical relevance especially in patients presenting short maxillary crowns or enlarged overbite values [31].

Considering the horizontal interproximal accuracy, retainers in the mandible (median 0.66 mm) were manufactured significantly more accurate than those in the maxilla (median 0.97 mm), obtaining a medium effect size (r = 0.41), which is supposed to reflect the more complex crown anatomy of palatal surfaces compared to lingual surfaces, the former often presenting more or less prominent marginal ridges with a large interindividual variety [2, 10, 15]. This difference was also visible in the comparison of the different retainer settings with median values ranging from 0.76 to 1.16 mm in the maxilla and 0.51 to 0.84 mm in the mandible, resulting in statistical significance with medium effect sizes for both arches. In both jaws, the manually bent retainers revealed the highest horizontal interproximal accuracy, while the CAD/CAM cut and CAD/CAM bent retainers showed comparable values with median differences ranging within 0.27 mm for the maxilla and 0.21 mm for the mandible, which is supposed to be clinically irrelevant [9, 26]. These findings are contradictory to the study of Kang et al. [8], who found CAD/CAM cut retainers to be the most accurate ones followed by manually bent and CAD/CAM bent retainers in both jaws, but reaching statistically significance only for the maxillary arch. In addition, the horizontal measurements obtained in the present study were found to be slightly larger than those reported by Kang et al. (absolute values were published only for the maxilla) [8]. Specifically, their study indicated a mean ± standard deviation of 0.59 ± 0.25 mm for maxillary CAD/CAM cut retainers, 0.71 ± 0.23 mm for manually bent retainers, and 1.01 ± 0.26 mm for CAD/CAM bent retainers. It is important to note that the reference study utilized a thinner CAD/CAM cut retainer, which probably led to higher accuracy [8]. Furthermore, the present investigation aimed to select maxillary models with a wide variety of tooth surface anatomies, which may have contributed to an increased level of inaccuracies.

Taking the location of the different interproximal areas into account, a statistically significant difference with a strong effect size was seen in the maxillary arch, indicating that the horizontal interproximal accuracy was higher between the incisors than between lateral incisors and canines. This finding is supported by the results of Koller et al. [9], who reported the same tendency when comparing the planned and achieved intraoral position of CAD/CAM cut retainers [9]. Given the fact that the 3–3 retainers in the maxilla are more prone to failure than shorter forms [6, 29] and the majority of wire breakages are located between the lateral incisors and canines [20], the present results support the synthesis of Zachrisson’s clinical experience to recommend 2–2 retainers for the maxillary jaw [30, 31].

Focusing on CAD/CAM bent retainers, the wire quality (3-stranded or 6‑stranded) had no statistically significant impact on either the vertical or on the horizontal interproximal accuracy. Nevertheless, the largest outliers in both dimensions (vertically: 1.81 mm, horizontally: 3.08 mm) were seen in the 3‑stranded retainers which had been planned using the OnyxCeph^®^ software. The direct comparison of the different planning software revealed contradictory results: while the vertical interproximal accuracy was higher for retainers planned with the OnyxCeph^®^ software, the FixR™ software led to more accurate horizontal interproximal results. However, since the underlying algorithms of the software used are not known, these results can only serve as an observation. As already mentioned above, the absolute difference of the medians, along with the weak effect size, puts the highly significant results of the Mann–Whitney test into perspective and suggests that, despite statistical significance, there is no clinical relevance of these differences.

## Conclusion

Considering the limitations of an in vitro study, the following conclusions concerning the interproximal accuracy of computer-aided design/computer-aided manufacturing (CAD/CAM) cut, CAD/CAM bent and manually bent retainers ranging from canine to canine can be drawn:

- Vertical interproximal accuracy was achieved best by CAD/CAM cut retainers, although the median vertical deviation of all manufacturing methods laid within a clinically acceptable range.
- Horizontal interproximal accuracy was highest in manually bent retainers. While all types of retainers seem clinically acceptable for their use in the mandible, maxillary retainers presented a larger variability especially in more distal interproximal areas.
- Focusing on CAD/CAM bent retainers, the wire quality (3-stranded/6-stranded) had no significant impact in either dimension. The use of different planning software (FixR™/OnyxCeph®) led to statistically significant, but clinically irrelevant differences in vertical and horizontal interproximal accuracy.

## Supplementary Information

**Supplementary table 1 **Dunn’s pairwise comparisons of vertical interproximal distance by setting adjusted to Hochberg. Black numbers indicate the respective *p*-values, blue numbers the effect size measure r

**Supplementary table 2 **Dunn’s pairwise comparisons of horizontal interproximal distance by setting adjusted to Hochberg. Black numbers indicate the respective *p*-values, blue numbers the effect size measure r

## Data Availability

The datasets used and/or analyzed during the current study are available from the corresponding author on reasonable request.
