# Supplementary material for: Interproximal accuracy of CAD/CAM machine cut, machine bent and manually bent retainers: An in vitro comparison
Source: J Orofac Orthop. 2025 Jul 30;87(6):567–80. doi: 10.1007/s00056-025-00602-0 (PMC13710456; doi:10.1007/s00056-025-00602-0)
Supplement: Supplementary file 1 [file 56_2025_602_MOESM1_ESM.docx]

**Supplementary table 1** Dunn´s pairwise comparisons of vertical interproximal distance by setting adjusted to Hochberg. Black numbers indicate the respective p-values, blue numbers the effect size measure r.

| **Maxilla** | 3-stranded FixR | 6-stranded FixR | 3-stranded Onyx | 6-stranded Onyx | 6-stranded manually |
| --- | --- | --- | --- | --- | --- |
| 6-stranded FixR | 0.4459 |  |  |  |  |
|  | 0.014 |  |  |  |  |
| 3-stranded Onyx | 0.7619 | 0.7787 |  |  |  |
|  | 0.088 | 0.101 |  |  |  |
| 6-stranded Onyx | 0.0118 | 0.0079 | 0.1294 |  |  |
|  | 0.314 | 0.328 | 0.226 |  |  |
| 6-stranded manually | 0.7322 | 0.6765 | 1 | 0.2168 |  |
|  | 0.116 | 0.13 | 0.029 | 0.198 |  |
| Memotain | 0.0168 | 0.0116 | 0.1735 | 0.8786 | 0.2731 |
|  | 0.299 | 0.312 | 0.211 | 0.015 | 0.182 |
| **Mandible** | 3-stranded FixR | 6-stranded FixR | 3-stranded Onyx | 6-stranded Onyx | 6-stranded manually |
| 6-stranded FixR | 0.1935 |  |  |  |  |
|  | 0.211 |  |  |  |  |
| 3-stranded Onyx | 0.6841 | 1 |  |  |  |
|  | 0.129 | 0.081 |  |  |  |
| 6-stranded Onyx | 1 | 0.759 | 0.6173 |  |  |
|  | 0.079 | 0.131 | 0.05 |  |  |
| 6-stranded manually | 1 | 0.6717 | 0.7925 | 0.4479 |  |
|  | 0.066 | 0.144 | 0.063 | 0.013 |  |
| Memotain | 0.0016 | 0.5589 | 0.0979 | 0.0241 | 0.017 |
|  | 0.37 | 0.159 | 0.24 | 0.29 | 0.303 |
