# Supplementary material for: Interproximal accuracy of CAD/CAM machine cut, machine bent and manually bent retainers: An in vitro comparison
Source: J Orofac Orthop. 2025 Jul 30;87(6):567–80. doi: 10.1007/s00056-025-00602-0 (PMC13710456; doi:10.1007/s00056-025-00602-0)
Supplement: Supplementary file 2 [file 56_2025_602_MOESM2_ESM.docx]

**Supplementary table 2** Dunn´s pairwise comparisons of horizontal interproximal distance by setting adjusted to Hochberg. Black numbers indicate the respective p-values, blue numbers the effect size measure r.

| **Maxilla** | 3-stranded FixR | 6-stranded FixR | 3-stranded Onyx | 6-stranded Onyx | 6-stranded manually |
| --- | --- | --- | --- | --- | --- |
| 6-stranded FixR | 1 |  |  |  |  |
|  | 0.067 |  |  |  |  |
| 3-stranded Onyx | 0.0323 | 0.0047 |  |  |  |
|  | 0.269 | 0.336 |  |  |  |
| 6-stranded Onyx | 0.0674 | 0.0122 | 0.3824 |  |  |
|  | 0.239 | 0.306 | 0.03 |  |  |
| 6-stranded manually | 0.1312 | 0.444 | 0 | 0.0001 |  |
|  | 0.202 | 0.135 | 0.471 | 0.441 |  |
| Memotain | 0.1387 | 0.032 | 0.7919 | 0.7399 | 0.0003 |
|  | 0.206 | 0.273 | 0.063 | 0.033 | 0.407 |
| **Mandible** | 3-stranded FixR | 6-stranded FixR | 3-stranded Onyx | 6-stranded Onyx | 6-stranded manually |
| 6-stranded FixR | 0.5596 |  |  |  |  |
|  | 0.108 |  |  |  |  |
| 3-stranded Onyx | 0.136 | 0.448 |  |  |  |
|  | 0.212 | 0.104 |  |  |  |
| 6-stranded Onyx | 0.0011 | 0.037 | 0.3319 |  |  |
|  | 0.379 | 0.271 | 0.167 |  |  |
| 6-stranded manually | 0.3744 | 0.0445 | 0.0017 | 0 |  |
|  | 0.153 | 0.262 | 0.365 | 0.533 |  |
| Memotain | 0.5945 | 0.4603 | 0.3475 | 0.0408 | 0.0379 |
|  | 0.118 | 0.01 | 0.094 | 0.261 | 0.272 |
